# Vascular Mimicry Expression in Invasive Ductal Carcinoma; A New Technique for Prospect of Aggressiveness

**DOI:** 10.30699/ijp.2019.94997.1939

**Published:** 2019-08-01

**Authors:** Amir Hossein Jafarian, Melika Kooshkiforooshani, Abdolshakor Rasoliostadi, Nema Mohamadian Roshan

**Affiliations:** 1 Department Of Pathology, Cancer Molecular Pathology Research Center, Faculty of Medicine, Mashhad University of Medical Sciences, Mashhad, Iran; 2 Pathology Department, School of Medicine, Mashhad University of Medical Sciences, Mashhad, Iran

**Keywords:** Vascular mimicry, Breast cancer, Immunohistochemistry staining

## Abstract

**Background & Objective:**

In vascular (vasculogenic) mimicry (VM), tumoral cells mimic the endothelial cells and form the extracellular matrix-rich tubular networks. It has been proposed that VM is more extensive in aggressive tumors. This study was designed to investigate the rate of VM expression in the stromal cells of invasive ductal carcinoma (IDC) and to find its relationship with other clinicopathological factors.

**Methods:**

In this cross-sectional study, 120 patients with histopathologic diagnosis of IDC who received mastectomy were included. The VM expression was determined by immunohistochemistry (IHC). The clinicopathologic data including age, tumor size, histological grade, clinical stage, axillary lymph node metastasis, hormonal receptors, and survival were documented.

**Results:**

The mean (±SD) age of the patients was 51 (±13.83) years old. The stromal VM expression was detected in 16 of 120 patients (13.3%). Twelve specimens (75%) of positive VM expression group had grade 3 which was higher than negative VM expression group (9 cases, 8.65%; *P*<0.001). The VM expression showed statistically significant relationship with higher histologic grade higher clinical stage (stage 3) of the tumor (62.5% vs. 87%; *P*=0.003), the presence of axillary lymph node metastasis (95.6% vs. 55.8%; *P*<0.001), and positive HER-2 (100% vs. 31.1%; *P*<0.001); but not estrogen receptor (ER) or progesterone receptor (PR). However, age, tumor size and mortality rate were not significantly different among the patients with and without VM expression.

**Conclusion:**

The stromal VM expression showed significant relationship with higher stage and grade of the tumor and the presence of nodal metastasis. The VM expression in IDC can be used as a marker for tumor aggressiveness.

## Introduction

Breast cancer is the most challenging malignancy in women which results in death in about one-fourth of the cases. Invasive (infiltrating) ductal carcinoma (IDC) is the most common form of breast cancer (80%) (1, 2).

To date, much effort has been done to identify the prognostic factors and their relevant therapeutic role in breast cancer (3). For instance, adequate blood supply is critical for tumor tissue to grow, proliferate, and disseminate hematogenously. For years, angiogenesis has been considered the sole mechanism of tumor vascularization (4). However, vascular (vasculogenic) mimicry (VM) is a novel pathologic marker for tumor aggressiveness. In VM, the formed bloodstreams are not lined by the endothelial cells. The VM expression has been observed in several tumors (such as gastric and breast cancer) with subsequent poorer prognosis and shorter survival (5).

The earlier reports regarding morphologic and molecular properties of VM focused on the melanoma in which the tumor cells co-expressed endothelial and tumor markers. The tumoral cells also formed laminin-rich structures with considerable collagens IV and VI. This creates a perfusion pathway for the rapidly growing tumors (6). The VM plays an important role in tumor growth, infiltration and metastasis. The tumor cells forming VM are a genetic reversion to a pluripotent embryonic-like genotype, a change called “cancer plasticity” (7). The VM expression has been reported in melanoma, ovarian cancer, inflammatory breast cancer, hepatocellular carcinoma, and soft tissue sarcomas. The VM expression has been confirmed to be an indicator of a poor prognosis in some malignant tumors. The patients with tumors demonstrating VM have a lower survival rate of about 5 year than patients having tumors without VM expression (8).

Sun and colleagues have shown that when endothelium-dependent vessels are inhibited by traditional anti-angiogenic therapies, the resulting hypoxia promotes VM formation to provide selective perfusion, leading to tumor aggression and metastasis (9). Another study revealed that VM with positive PAS and negative CD31 is lined by tumor cells and independent of epithelial cells. As a unique perfusion way, VM has been observed in a variety of aggressive tumors. However, their specific roles in VM still remain unclear and PR, ER, HER2 markers have not been studied (10). Guo et al. in a systematic meta-analysis showed that VM expression was associated with a poor prognosis in the patients with gastric cancer (11).

Since predicting breast cancer progression and significant related histopathologic predictors are clinically important, this study intended to investigate the role of VM in IDC and its relationship with clinicopathological factors.

## Materials and Methods

The population of this cross-sectional study consisted of 120 female patients with the diagnosis of IDC who underwent mastectomy at our Medical Center from March 2011 to February 2016.‌ The inclusion criteria were complete information of the required clinicopathological factors including axillary lymph nodes and IHC for PR (progesterone receptor), ER (esterogen receptor), and HER2 (human epidermal growth factor receptor 2).

HER2 positive cases were defined as those with IHC score of 3+ (strong membrane staining of more than 10% cells) or 2+ confirmed by Fluorescence In Situ Hybridization (FISH) or Chromogenic In Situ Hybridization (CISH).

A checklist of personal and paraclinical information, past medical history and prognosis was provided. Subsequently, hematoxylin and eosin (H&E)-stained slides of all cases were checked. The paraffin blocks with sufficient tumoral and stromal tissues were taken and two pathologists checked and confirmed the IHC of the samples to identify the expression of vascular mimicry. The gathered variables include age, tumor size, tumor grade, tumor stage, prognosis, PR, ER, HER2 receptors, axillary lymphadenopathy and vascular mimicry. Finally, the data was normalized and statistically analyzed using SPSS software.

Statistics

Descriptive statistics were used to express data analysis. In order to compare qualitative data between two groups with and without VM expression, the Chi-square test was used. The continuous data were compared between the two groups using the Student *t-*test. The significance level was set at 0.05. All analyses were performed using SPSS software (ver. 16.0, IBM).

Ethics

The study protocol was fully supported by the Research Council Ethics Committee of our Medical University. No informed consent was required and mastectomy was indicated for the patients. The study was in conformity with the Declaration of Helsinki.

## Results

The VM expression was detected in 16 of 120 specimens (13.3%). The mean (±SD) age in VM positive group was 54.31 (±10.9) years old which was not significantly different from those without VM expression (51.19±11.6 years old; *P*=0.156). Twelve specimens in positive VM expression group had grade 3 (12 of 16 specimens, 75%) which was significantly higher than specimens with negative VM (9 of 104 specimens, 8.65%; *P*<0.001). Table 1 shows the comparison of tumor size, stage, grade, and lymphadenopathy between the two groups of patients with and without VM expression.

HER2 receptor was positive in all specimens with VM expression (16 specimens, 100%) which was significantly higher than the group with negative VM expression (32 specimens, 31.1%; *P*<0.001). But, only one specimen in each group with ER+ and PR+ showed VM expression (Table 2).

Mortality

At follow-up, five patients of 16 patients (31.25%) with VM-positive tumors died. In the group with VM-negative tumors, 24 patients (23.1%) died (*P*=0.114).

**Table 1:** Comparison of the clinicopathological factors between two groups of female patients with invasive ductal carcinoma according to the vascular mimicry expression

		Positive VM expression (N= 16)	Negative VM expression (N= 104)	*P* value
Tumor size	< 3 cm	7 (43.75%)	45 (43.26%)	0.146
	≥ 3 cm	9 (56.25%)	59 (56.73%)	
Tumor histological grade	1	0	50 (48.07%)	<0.001
	2	4 (25%)	45 (43.26%)	
	3	12 (75%)	9 (8.65%)	
Clinical stage	1	0	39 (37.5%)	0.003
	2	6 (37.5%)	54 (51.92%)	
	3	10 (62.5%)	11 (10.5%)	
Axillary lymph node metastasis		14 (87.5%)	58 (55.76%)	<0.001

**Table 2:** Comparison of the hormonal receptors between two groups of female patients with invasive ductal carcinoma according to the vascular mimicry (VM) expression

	Positive VM expression (N=16)	Negative VM expression (N=104)	*P* value
Positive ER	1 (6.25%)	61 (58.65%)	<0.001
Positive PR	1 (6.25%)	71 (68.26%)	<0.001
Positive HER2	16 (100%)	32 (31.1%)	<0.001

ER: estrogen receptor; PR: progesterone receptor; HER2: human epidermal growth factor receptor 2

## Discussion

VM, a newly defined pattern of tumor blood supply has been observed in aggressive tumor cells that result in formation of structures which resemble the vessels (12). In addition to the ability of VM in making tumors more aggressive, efforts are done to recognize the signaling pathways in VM formation and consequently development of new anti-tumor agents (13). To evaluate the correlation of those traditional information and VM in human breast cancer, we examined VM expression using IHC in 120 breast cancer specimens. According to our findings, 13.3% of IDC specimens expressed VM. Studies on the rate of VM expression in breast cancer are not sufficient. The current published reports have noted VM expression rate as low as 7.9% (5) to 24% (14). No statistically significant difference was observed between those who expressed VM and those with no VM expression in terms of age, tumor size, and mortality. On the other hand, histological grade and clinical stage of breast cancer, axillary lymph node metastasis, and positive HER2 had significant relationship with VM expression. These findings were in accordance with several similar studies (5). 

Liu and colleagues (15) observed VM formation in 22.5% of invasive breast carcinoma specimens. Compatible with our findings, they showed significant relationship between VM expression and lymph node metastasis and higher stage of the disease. Notably, they found that the rate of positive VM expression increased in parallel to HER2 expression, which is in agreement with our results. Similarly, the median number of VM channels was greatest in the HER2 3+ tumors, indicating that HER2 contributes to VM formation in breast cancer. The overexpression of HER2 in breast cancer is associated with poorer outcomes including shorter survival times. The mentioned study (15) did not refer to the association of VM expression with ER and PR status. The positive rates of ER and PR in VM-positive specimens were significantly lower than VM-negative patients.

In a recent review of eight studies, the pooled prevalence rate of positive VM in breast cancer was reported at 24% with significant association with lymph node metastasis as well as larger tumor size. The authors also found that VM-positive patients had a shorter survival rate (14). Although this study provided important data regarding the role of VM positivity in breast cancer, however, further studies are needed as evidence is not sufficient in the literature to reach a solid conclusion. In the current study, no significant difference was found between mortality and VM-positive and VM-negative patients. As angiogenesis is one of the established factors in breast cancer dissemination and transforming in situ epithelium to more invasive form (5, 16), it is presumed that VM may have association with more aggressive form and mortality. According to our results, as we only included patients with IDC breast cancer, the VM expression was associated with a higher histological grade of the tumors, but not with mortality. This can be justified as different clinicopathological factors contribution to mortality. 

This study included a considerable number of patients with IDC to perform more precise estimation of VM-positive rate. In addition, several clinicopathological factors were investigated to determine any relationship with positive VM expression. However, we had some limitations. We were not able to document overall survival (OS) time.

## Conclusion

We observed VM expression in 13.3% of IDC specimens. The VM expression showed significant relationship with histological grade, tumor stage, axillary lymph node metastasis, and positive HER2. However, VM expression did not exhibit any relationship with survival, age, or tumor size.
